# Defining the relationship between gastroesophageal reflux and cough: probabilities, possibilities and limitations

**DOI:** 10.1186/1745-9974-3-4

**Published:** 2007-03-20

**Authors:** Matthew M Eastburn, Peter H Katelaris, Anne B Chang

**Affiliations:** 1 Department of Respiratory Medicine, Royal Children's Hospital, Brisbane, Australia; 2 School of Information Technology and Electrical Engineering, University of Queensland, St Lucia, Queensland, Australia; 3 Department of Gastroenterology, University of Sydney, Concord Hospital, Sydney, Australia; 4 Child Health Division, Menzies School of Health Research, Darwin, Northern Territory, Australia

## Abstract

The common co-existence of cough and gastroesophageal reflux disease (GORD) is well established. However, ascertaining cause and effect is more difficult for many reasons that include occurrence by chance of two common symptoms, the changing definition of GORD, equipment limitations and the lack of randomised controlled trials. Given these difficulties, it is not surprising that there is disparity of opinion between respiratory and gastroenterology society guidelines on the link between GORD and chronic cough. This commentary explores of these issues.

## Background

The first guideline on the management of cough championed by Irwin [1] made a significant positive impact. Not surprisingly other guidelines on chronic cough [2-5] have since been published. American [2], European [3] and British [5] respiratory guidelines for the management of chronic unexplained cough in adults advocate empirical treatment of gastroesophageal reflux disease (GORD) with a variety of medications including proton pump inhibitors (PPIs). In contrast, guidelines from some national gastroenterological societies are less definitive about the association between cough and GORD [6-9] Paediatric cough guidelines do not favour the empirical approach in adults because GORD as a cause of isolated cough is rare in children [10,11]. Is there evidence for a true difference or do these differences exist because opinion leaders in their respective fields have different views? In this commentary, important limitations in understanding the association between cough and GORD are explored.

That cough and GORD commonly coexist is indisputable in both children [12] and adults [13]. The questions of whether this is 'cause and effect' [14], 'whether GORD causes cough or vice versa' [15] and 'how commonly can the symptom of cough be attributed to GORD' remain controversial [9,16]. Nevertheless the problem is real; in the community the burden of cough and GORD, in isolation or in combination, is high. Chronic cough is associated with significant morbidity [17] and the economic cost in terms of medications alone, is billions of dollars [18]. Empirical acid antisecretory treatment of cough in adults adds to this cost. In Australia alone, where the costs of medications are heavily subsided by the government, three PPIs are in the top 10 drugs by cost [19]. In 2006 these 3 PPIs alone costs the Australian tax payers almost A$42.5 million [19].

## Cough and reflux: two common symptoms, chance occurrence and which came first?

In many developed countries, cough is the most common symptom presenting to doctors [18,20]. Chronic cough can affect up to 20% of the population [21] whilst the prevalence of GORD in Western populations is up to 25% [22]. Thus the upper limit of probability of a chance association as independent events is 5% of the population. In selected patient cohort studies the higher prevalence of these symptoms would increase the likelihood of a chance occurrence. That is, in a cohort study of subjects with chronic cough, the chance occurrence of GORD as an independent event may be up to 25%.

Not only is it possible that two common symptoms may coexist merely by chance but determining which symptom came first is difficult and opinions vary. Acutely, cough can precipitate reflux events as shown objectively by Paterson and Murat [23] with cough bursts defined on oesophageal manometry. Using ambulatory pressure-pH-impedance monitoring, Sifrim and colleagues reported that the majority (69.4%) of cough events in subjects with chronic cough, were considered independent of reflux, whereas 30.6% occurred within two minutes of a reflux episode [24]. In a review using strict definitions, Dent and colleagues found that "in the year following the diagnosis of GORD, patients were at increased risk of a first time diagnosis of cough (OR 1.7, 95% CI 1.4–2.1), angina (OR 3.2, 95% CI 2.1–4.9), gall bladder disease (OR 3.7, 95% CI 2.1–6.7), sinusitis (OR 1.6, 95% CI 1.2–2.0) and chest pain (OR 2.3, 95% CI 1.8–2.8) [25]. However, despite the reported frequency of assumed cough from GORD and the common clinical observation that treatment for GORD may lead to resolution of cough, at least in some people [26,27] there is glaring lack of published randomised trials [28].

## Differences between respiratory and gastroenterology society publications

There is a degree of variance between adult respiratory and gastroenterological society guidelines when considering a possible association between airway symptoms and GORD. While gastroenterological society publications have been more cautious in linking upper airway symptoms to GORD [6-9,29], adult respiratory ones largely endorse the cause and effect [5,30]. Recent gastroenterology society recommendations are based largely on systematic reviews and meta-analysis [9,31]. In contrast, the latest published cough guideline [5] omitted meta-analysis data [27,28] which had similar findings to the approach adopted by gastroenterology societies [6-9,29].

## Defining GORD- the changing goal posts

Reflux of gastric contents into the oesophagus can be acidic, weakly acidic or weakly alkaline (non acid reflux) and includes 'volume' reflux. Prior to the description of non-acid or weakly acidic reflux, proponents that cough is commonly caused by GORD have described that almost all (>75%) cough (if not all) was associated with acidification of the oesophagus and/or resolved with acid suppression therapy [32-34]. However, non-acid reflux can now be measured using multi-channel intraluminal impedance combined with pH monitoring and has been shown to be associated with an undefined but significant proportion of GORD associated cough [24]. Indeed, until the last 12 months pH monitoring for acid reflux was the recommended standard for defining cough associated with GORD with published positive and negative predictive values of 89 and 100% respectively [2,3]. However, the predictive value of pH monitoring has been questioned even for the diagnosis of GORD itself [35] with no agreement about the gold standard for the diagnosis of GORD [6]. Furthermore, while the definition of abnormal acidification has been largely agreed, with three age dependent cut-offs [6,8], such definitions for associating GORD to cough remains indeterminate. The belief in adult respiratory practice, that cough related to GORD may occur without any reflux symptoms [5] results in frequent empirical therapy for any patient with chronic cough with or without GORD symptoms. However, a recent international consensus statement (The Montreal Delphi consensus report) following a review of the literature concluded that unexplained laryngeal and respiratory symptoms were unlikely to be related to GORD in the absence of heartburn or regurgitation and that typical heartburn and regurgitation are highly specific for GORD [9].

## Equipment limitations

**Figure 1:**
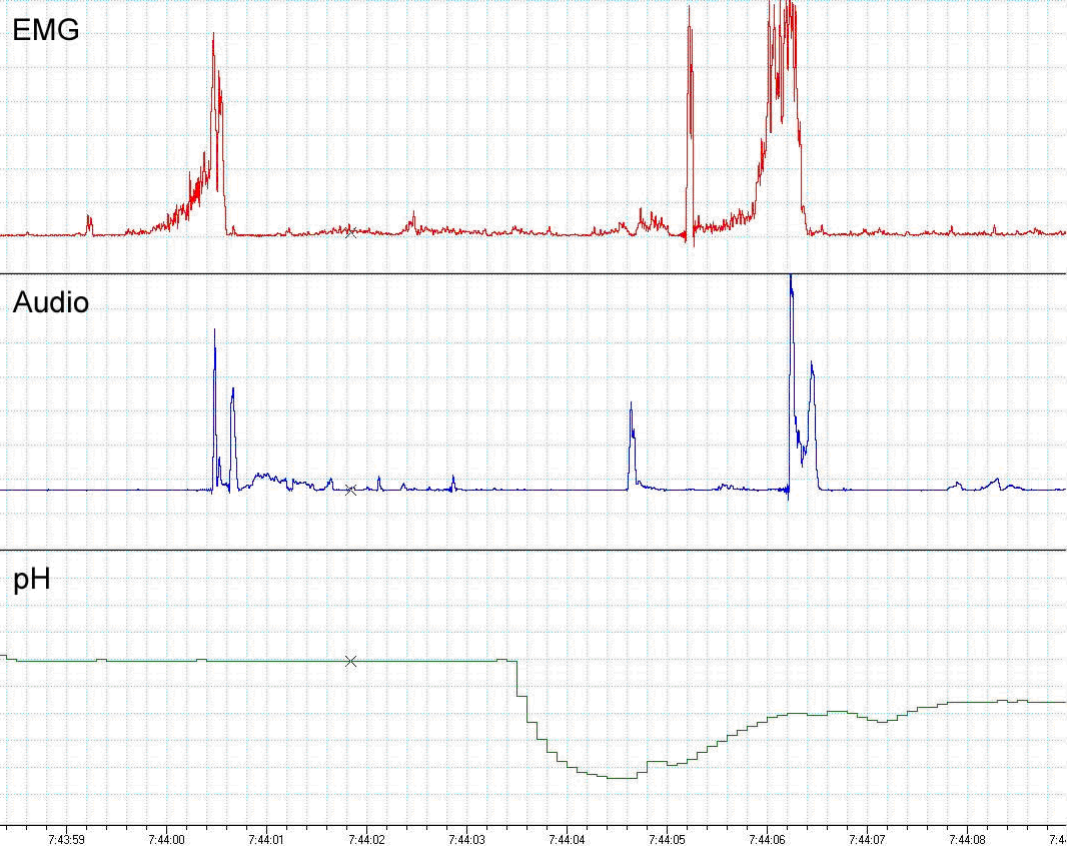
Cough preceding a pH drop followed by another cough. Recordings from a specifically built pHmetry-cough-logger with a capture rate of 10 Hz (40 times the commercially available systems). Time scale in hours:mins:secs.

Almost all (if not all) commercial pHmetry systems have a maximum capture or download rate of 0.25 Hz. That is, data points are recorded once every 4 seconds. The active respiratory muscle phase of a single cough epoch lasts 0.6–0.8 secs (figure 1) and the glottic closure phase of cough whereby the greatest intrathoracic pressure is generated lasts 0.2 secs [36]. Thus an objective study of cough and reflux would require a capture rate of at least 5 Hz (one data point every 0.2 secs) to ascertain if cough occurs before a reflux event. Subjective scoring or event marking on a commercial system is highly inaccurate as far as timing is concerned, so it is not possible to know whether a cough occurs before a pH drop (or vice versa) when data is captured once every 4 secs. Furthermore, data captured on synchronised (for example to the nearest second) separate instruments as opposed to a single time frame will give erroneous results given that resolution rates has to be less than the compressive phase of a cough when intrathoracic pressures peaks up to 300 mmHg [36] as it is the phase most likely associated with a reflux event. This is illustrated in figure 1 obtained using a specifically built pHmetry-cough-logger with a capture rate of 10 Hz (ie 10 data points every one sec).

Sifrim and colleagues [24] used manometry and pHmetry (at a slow capture rate 0.25 Hz) to define the occurrence of acid reflux to cough (in addition to other data). However, 'cough' was defined on manometry data and current manometry labelling of 'cough' has only been partially validated when compared to how cough loggers are validated [37,38]. Physiologically, changes seen in oesophageal manometry reflect intra-thoracic changes and thus events such as sneeze, hiccups, throat-clearing manoeuvres would appear similar to coughs, as shown in respiratory muscle EMG changes. Thus, it is likely that the association reported was over-estimated.

## Acute vs chronic data: are they related?

In addition to the above, it is unknown if acute effects related to cough preceding or following a reflux event is relevant to the management of chronic cough. In view of equipment limitations, it is not surprising that there are no publications on this, or any controlled trials. It is biologically possible that cough takes a longer time to resolve following treatment compared to typical reflux symptoms if there is up-regulation of cough neuro-pathway [39] that may take time to re-equilibrate. However in Ours and colleagues randomised controlled trial, the 'time to response' was 2 weeks [26].

## Laryngo-pharyngeal reflux (LPR)?

Ear, nose and throat (ENT) diseases and LPR are widely regarded as a cause of chronic cough related to GORD. However, all controlled trials to date where subjects were enrolled from ENT clinics and cough was an outcome measure have shown that GORD treatment is not efficacious when compared to placebo [28]. Two additional controlled studies since a comprehensive review [16] also showed that neither PPI nor fundoplication were efficacious [40,41]. The former was the largest study involving GORD therapy with cough as an outcome (n = 145). In contrast, "uncontrolled studies suggest that 40–100% of patients who have suspected acid-related ENT symptoms improve on aggressive anti-reflux therapy" [16].

## The way forward?

Consumers and medical practitioners may be content to resort to a therapeutic trial for at least 3 months in all cases of chronic unexplained cough, as suggested by some [5]. Despite the apparent convenience of such an approach, it is not without risk of adverse events [42,43], incurs significant costs and is contrary to the emerging evidence that suggests that this strategy will meet with infrequent success particularly when cough is not associated with typical reflux symptoms. The advice of Bourke and Drumm (when discussing the history of the use of cisapride for GORD) advocated that guidelines must be multidisciplinary, based on systematic review of published work, and should explicitly link recommendations to the supporting evidence, is pertinent despite the excellent safety record of PPIs [44].

Further study of this relatively common clinical conundrum is clearly required. High quality placebo controlled randomised trials using a combination of objective and subjective outcomes in both adults and children [28] are needed. Furthermore, better characterisation of the predictive value of clinical features and measurable abnormalities of GORD associated with cough will result in better selection of patients for therapeutic trials of PPIs or other therapies. Moreover, the duration of therapy and time to response needs to be better defined as advice by some that treatment for cough associated with reflux can take up to one year is impractical. Lastly, to accurately and definitively relate cough to pH change temporally, it may be necessary to have an instrument with a sufficiently fast capture, recording rate and response time to allow more precise data collection, something that is lacking in currently available commercial pHmetry recorders for studies relating to cough.

## Conclusion

The common co-existence of cough and GORD is well established. By chance alone the occurrence of these as independent events may be as high as 5% of the general population. Ascertaining cause and effect is however more difficult. Although some patients may have resolution of chronic cough with therapies for GORD there is still insufficient evidence to determine whether GORD is a common cause of chronic cough. A multi-disciplinary approach involving respiratory physicians, gastroenterologists and ENT surgeons is required to better define this association and to promulgate consistent guidelines based on the best evidence. Until randomised placebo controlled clinical trials are performed with adequate power and using adequate instrumentation guidance for therapy in clinical practice will remain based on sub-optimal evidence and this conundrum will remain unresolved.
